# Evolution of a Lung-Sparing Strategy with Sleeve Lobectomy and Induction Therapy for Non-small Cell Lung Cancer: 20-Year Experience at a Single Institution

**DOI:** 10.1007/s00268-015-3330-z

**Published:** 2015-12-28

**Authors:** Tetsuzo Tagawa, Takekazu Iwata, Takahiro Nakajima, Hidemi Suzuki, Shigetoshi Yoshida, Ichiro Yoshino

**Affiliations:** Department of General Thoracic Surgery, Graduate School of Medicine, Chiba University, 1-8-1 Inohana, Chiba, 260-8670 Japan

## Abstract

**Background:**

To elucidate the evolution of a lung-sparing strategy with sleeve lobectomy (SL) and induction therapy for non-small cell lung cancer (NSCLC).

**Methods:**

We retrospectively reviewed 205 patients with NSCLC who underwent pneumonectomy (PN, *n* = 54) or SL (*n* = 151) from 1994 to 2013. The study period was divided into four 5-year periods, and surgical trends were analyzed, focusing on the PN:SL ratio.

**Results:**

PN was associated with a significantly advanced pathological stage, a larger tumor size and less pulmonary function compared with SL. The PN group had higher 30-day (3.7 vs. 0 %, *p* = 0.018) and 90-day (13.0 vs. 1.3 %, *p* = 0.0003) mortality than the SL group. The overall 5-year survival rate was significantly higher with SL (71.5 %) versus PN (42.8 %, *p* = 0.011) for patients with pN0–1. The ratio of PN among total surgeries decreased significantly over the four periods (1994–1998, 1999–2003, 2004–2008, and 2009–2013) from 5.63 % to 3.17, 1.40, and 1.38 %, respectively (*p* < 0.0001); in contrast, the PN:SL ratio increased significantly from 1.64 to 2.50, 3.71, and 5.44, respectively (*p* = 0.041). During the last period, when we introduced induction therapy, 38 of 651 who received surgery underwent induction therapy. The PN:SL ratios of those who did and did not undergo induction therapy were 15 (PN: 1, SL: 15) and 4.25 (PN: 8, SL: 34), respectively.

**Conclusions:**

A lung-sparing strategy with SL for NSCLC can decrease the PN rate to less than 2 % with less mortality. Induction therapy may facilitate SL and increase the PN:SL ratio.

## Introduction

The purpose of surgical resection for non-small cell lung cancer (NSCLC) is to achieve complete resection of the tumor and lymph nodes to maximize the possibility of cure. Bronchial and/or pulmonary arterial sleeve lobectomy (SL) is a lung parenchyma-sparing procedure that aims for complete resection of tumors invading the central structures. SL was originally indicated for patients with reduced pulmonary function, who were intolerant of pneumonectomy (PN). However, SL is now indicated even for patients with sufficient pulmonary function to avoid PN, which causes substantial loss of pulmonary function and thus quality of life. Much evidence has been accumulated that the long-term survival after SL is favorable to that of PN, with lower mortality and morbidity [1–7]. A recent meta-analysis by Shi et al. demonstrated that SL provides lower mortality, better long-term survival, less loss of function, and better quality of life than PN without increasing morbidity and locoregional recurrences [8]. These results have encouraged surgeons to pursue lung parenchyma-sparing strategies more aggressively [2, 9–12]. Gómez-Caro and colleagues have reported their aggressive policy of avoiding PN by determining the appropriate PN to SL (PN:SL) ratio, which might reflect the institutes’ lung-sparing policy [13].

Induction therapy has been increasingly used for locally advanced NSCLC to downstage tumors and to facilitate complete resection. However, very few studies have compared outcomes of SL and PN after induction therapy [14, 15]. Maurizi et al. reported that SL represented a valid therapeutic option even after induction therapy, providing better long-term survival than PN with no increase in morbidity or recurrence [14]. Rendina and coworkers suggested that induction therapy may facilitate SL, reducing the need for PN while maintaining the completeness of resection [16].

We perform SL to avoid PN whenever it is technically and oncologically feasible, even for patients with sufficient pulmonary function. In this retrospective study, we reviewed the outcomes of PN and SL in our institute and elucidated the evolution of a lung-sparing strategy to avoid PN with SL and induction therapy.

## Materials and methods

We retrospectively reviewed the clinical records of 205 patients with NSCLC who underwent PN (*n* = 54) or SL (*n* = 151) at our institution from January 1994 to December 2013. During the same period, 2047 patients with NSCLC underwent surgery at our institution. The 20-year study period was then divided into four periods of 5 years each, and the trends in surgical strategy were analyzed, focusing on the PN:SL ratio. The Ethics Committee of Chiba University approved this study and granted a waiver for patient consent.

The preoperative work-up for all patients included physical examination, chest radiography, contrast-enhanced thoracic, upper-abdominal computed tomography (CT), cerebral CT or magnetic resonance imaging, and isotonic bone scanning. Positron emission tomography ([^18^F]-FDG-PET) was frequently used in recent years. Bronchoscopy was performed to confirm the diagnosis of malignancy and to observe the extent of tumor invasion to the bronchus. Suspected hilar and mediastinal lymph nodal involvements by CT or FDG-PET were confirmed pathologically by endobronchial ultrasound-guided transbronchial needle aspiration [17]. Patients were staged according to the TNM classification before the treatment. In this study, all patients were reassessed with the new 7th TNM edition. From 2008, induction therapy, especially chemoradiotherapy, was introduced for the patients with central tumors or tumors with pathologically positive mediastinal nodal involvement. All induction chemotherapy and chemoradiotherapy protocols were platinum based, but were combined with a variety of other agents: oral S-1 in 14 patients, gemcitabine in 2 patients, paclitaxel in 2 patients, paclitaxel plus bevacizumab in 2 patients. Radiotherapy was given concurrently with chemotherapy.

Surgical resection was performed using standard surgical techniques with dissection of systematic hilar and mediastinal lymph nodes. SL was performed whenever technically and oncologically feasible, even for patients with sufficient pulmonary function. Frozen sections of the resected bronchial or vascular margins were always examined to ensure complete resection. The bronchial stump was routinely covered with a pedicled flap of pericardial fat or intercostal muscle in patients who underwent bronchial SL. Resection was defined as complete (R0) if all gross disease was removed and if all surgical margins were free of tumor cells. Incomplete resection (R1 and R2) indicated that surgical margins were microscopically positive (R1) or macroscopically positive (R2).

Patient demographics were compared between groups by *t* test for continuous variables (mean and standard deviations) and *χ*^2^ analysis for categorical variables (frequency and percentages) as appropriate. Survival was calculated from the date of surgery until the date of death (due to any cause) or last follow-up (censored). Survival curves were created using the Kaplan–Meier method, and statistically significant differences between survival curves were examined using log-rank tests. A *p* value derived from two-tailed tests of less than or equal to 0.05 was considered significant. All data were analyzed using JMP, version 5.0 (SAS Institute, Inc., Cary, NC).

## Results

### Patient characteristics

Patients’ clinical characteristics according to the type of surgery (PN or SL) are shown in Table 1. There were no significant differences in age, sex, histology, and smoking status between the groups. However, patients who underwent PN had a significantly advanced pathological stage, advanced pathological lymph nodal status, and a larger tumor size than those who underwent SL. In terms of pathological stage, 44 of 54 patients (81 %) in the PN group were stage III–IV, while 60 of 151 patients (39 %) in the SL group were stage III–IV. The preoperative FEV1 of PN group was significantly worse than that of SL group. This is because we perform SL to avoid PN even for patients with sufficient pulmonary function. Both groups achieved high complete resection rate. Eighteen of 151 (12 %) patients in the SL group received induction therapy, consisting of chemoradiotherapy in 16 patients and chemotherapy in 2 patients, while only 2 of 54 (4 %) patients in the PN group had induction chemotherapy (*p* = 0.057). Three patients (5.6 %) in the PN group and 31 patients (20.5 %) in the SL group were eligible for adjuvant therapy (*p* = 0.028).

**Table 1:** Patient characteristics

Variables	PN (n = 54)	SL (*n* = 151)	*p*
	*n* (%)	*n* (%)	
Age (mean ± SD)	62.8 ± 8.9	63.8 ± 9.3	0.40
Sex			
Male	42 (78)	122 (81)	0.64
Female	12 (22)	29 (19)	
Smoker	44 (81)	116 (77)	0.76
Histologies			0.85
Adenocarcinoma	17 (31)	45 (30)	
Squamous cell carcinoma	31 (57)	84 (56)	
Large cell carcinoma	2 (4)	8 (5)	
Other	4 (7)	14 (9)	
Tumor diameter (mm)			0.0055*
Median (range)	55 (20–180)	42 (7–113)	
Tumor side			
Right/left	25/29	84/67	
Preoperative FEV_1_ (L) (mean ± SD)	2.05 ± 0.64	2.27 ± 0.65	0.04*
Preoperative FEV_1_% (%), (mean ± SD)	74.2 ± 9.51	73.1 ± 9.84	0.45
pStages			0.0001*
IA/IB	2 (4)	43 (28)	
IlA/IlB	7 (13)	47 (31)	
IIIA/IIB	39 (72)	55 (36)	
IV	5 (9)	5 (3)	
Unknown	1 (2)	1 (1)	
pNodal status			0.0001*
N0	6 (11)	60 (40)	
N1	14 (26)	47 (31)	
N2	30 (56)	41 (27)	
N3	2 (4)	2 (1)	
Unknown	2 (4)	1 (1)	
Induction therapy	2 (4)	18 (12)	0.057
Adjuvant therapy	3 (6)	31 (21)	0.028*
Completeness of resection			0.18
Complete resection	51 (96)	141 (93)	
Incomplete resection	2 (4)	10 (7)	

*PN* pneumonectomy, *SL* sleeve lobectomy, *SD* standard deviation, *FEV*_*1*_ forced expiratory volume in 1 s

* Statistically significant

Table 2 shows the types of SL and lung resection that were performed. The most frequent procedure was bronchial SL (*n* = 74, 49 %), followed by vascular SL (*n* = 43, 28.5 %) and broncho-vascular SL (*n* = 34, 22.5 %). Regarding lung resection, the right upper lobe was the most frequent area resected (*n* = 52, 34.4 %). Extended SL resection involving more than one lobe was achieved in 27 patients (17.9 %).

**Table 2:** Types of SL and lung resection

Lung resection	Bronchial SL (*n* = 74)	Vascular SL (*n* = 43)	Broncho-vascular SL (*n* = 34)	Total (*n* = 151)
RUL	36	4	12	52
RML	4	0	0	4
RLL	9	1	0	10
RUML	2	2	1	5
RMLL	3	3	2	8
RUL + S6	1	1	2	4
RUML + S6	0	0	1	1
LUL	6	28	7	41
LLL	9	3	5	17
LUL + S6	0	1	2	3
LLL + S4/5	4	0	2	6

*SL* sleeve lobectomy, *RUL* right upper lobe, *RML* right middle lobe, *RLL* right lower lobe, *RUML* right upper and middle lobes, *RMLL* right middle and lower lobes, *S6* superior segment, *LUL* left upper lobe, *LLL* left lower lobe, *S4*/*5* lingular segment

### Mortality and morbidity

Table 3 shows postoperative mortality and morbidity. Patients who underwent PN had significantly higher 30-day (3.7 vs. 0 %, *p* = 0.018) and 90-day (13.0 vs. 1.3 %. *p* = 0.0003) mortality rates than those who underwent SL. Two of 54 patients in the PN group died within 30 days: 1 patient died from a myocardial infarction followed by acute respiratory distress syndrome (ARDS), and the other died from a bronchial fistula with empyema followed by ARDS. An additional five patients in the PN group died within 90 days from ARDS in two patients, interstitial pneumonia in one patient, and tumor recurrence in two patients. Two of 151 patients in the SL group died within 90 days: one patient died from a bronchial fistula with empyema, and the other died from pneumonia. In terms of morbidity, there were no statistically significant differences in the rate of major (16.7 vs. 9.1 %, *p* = 0.19) and minor (25.9 vs. 23.2 %, *p* = 0.68) complications between groups.

**Table 3:** Mortality and morbidity

Variables	PN (*n* = 54)	SL (*n* = 151)	*p*
	*n* (%)	*n* (%)	
30-day mortality	2 (3.7)	0	0.018*
90-day mortality	7 (13.0)	2 (1.3)	0.0003*
Major complications	9 (16.7)	15 (9.1)	0.19
Broncho-pleural fistula	3 (5.6)	6 (4.0)	0.62
Empyema	3 (5.6)	8 (5.3)	0.30
ARDS	5 (9.3)	4 (2.6)	0.052
Postoperative bleeding	1 (1.9)	0	0.094
Recurrent nerve palsy	0	1 (0.66)	0.55
Gastrointestinal hemorrhage	0	1 (0.66)	0.55
Myocardial infarction	2 (3.7)	1 (0.66)	0.12
Minor complications	14 (25.9)	35 (23.2)	0.68
Arrhythmia	12 (22.2)	23 (15.2)	0.24
Pneumonia	3 (5.6)	20 (13.2)	0.12

*PN* pneumonectomy, *SL* sleeve lobectomy, *ARDS* acute respiratory distress syndrome

* Statistically significant

### Survival

**Fig. 1:**
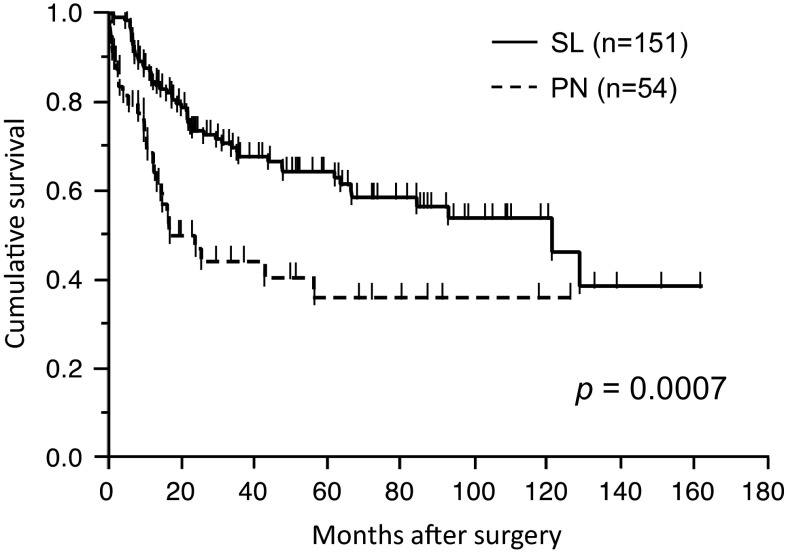
Overall survival of 151 patients who underwent sleeve lobectomy (SL) and 54 patients who underwent pneumonectomy (PN). The 5-year survival rate was 62.6 versus 35.6 % with SL and PN, respectively (*p* = 0.0007)

**Fig. 2:**
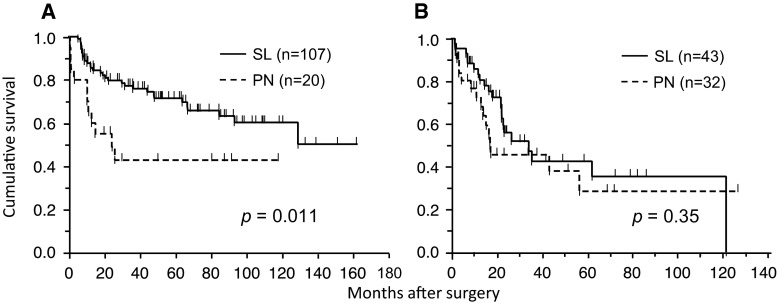
**a** Overall survival of 107 patients who underwent SL and 20 patients who underwent PN with pN0–1. The 5-year survival rate was 71.5 and 42.8 % with SL and PN, respectively (*p* = 0.011). **b** Overall survival of 43 patients who underwent SL and 32 patients who underwent PN with pN2–3. The 5-year survival rate was 42.6 and 28.5 % with SL and PN, respectively (*p* = 0.35)

The median follow-up time for the 54 patients in the PN group was 27.2 months (range 0.6–126.5 months), while that for the 151 patients in the SL group was 44.2 months (range 0.4–162 months). The overall 5-year survival rate of the patients in the SL group was significantly higher than that of the patients in the PN group (62.6 vs. 35.6 %, respectively; *p* = 0.0007, Fig. 1). Part of this difference was explained by the significantly advanced pathological stage, advanced pathological lymph nodal status, and higher postoperative mortality of the patients in the PN group. Among patients with pN0–1 disease, the 5-year survival rate after SL (*n* = 107) and PN (*n* = 20) was 71.5 and 42.8 %, respectively (*p* = 0.011, Fig. 2a). In contrast, there was no difference in survival between SL (*n* = 43) and PN (*n* = 32) for patients with pN2–3 (42.6 vs. 28.5 %, respectively; *p* = 0.35, Fig. 2b). In terms of pathological stage, there was no survival difference between groups for patients with stage I–II (*p* = 0.34) and stage III–IV (*p* = 0.23).

### Recurrence

Data were available for only 107 patients (19 patients in the PN group and 88 patients in the SL group) who underwent surgery from 2003 to 2013. Eight of 19 patients in the PN group (42.1 %) had recurrence, consisting of systemic recurrence in 5 patients (26.3 %), local recurrence in 2 patients (10.5 %), and both systemic and local recurrence in 1 patient (5.3 %), while 38 of 88 patients (43.2 %) in the SL group had recurrence, consisting of systemic recurrence in 23 patients (26.1 %), local recurrence in 10 patients (11.4 %), and both systemic and local recurrence in 5 patients (5.7 %).

### Trends in surgical procedures and the PN:SL ratio

Table 4 shows the trends in surgical procedures during the 20-year study period, which was divided into four periods of 5 years each. The ratio of PN among total surgeries decreased significantly over the four periods (1994–1998, 1999–2003, 2004–2008, and 2009–2013) from 5.63 % to 3.17, 1.40, and 1.38 %, respectively (*p* < 0.0001). The ratio of SL among total surgeries also decreased from the first (1994–1998) to the third (2004–2008) period from 9.20 to 7.94 and 5.20 %. These declines may reflect the increase of small peripheral lung cancer in recent years. However, the ratio of SL increased in the last period (2009–2013) to 7.53 %. The PN:SL ratio increased significantly over the four periods from 1.64 to 2.50, 3.71, and 5.44, respectively (*p* = 0.041) and the average was 2.80. During the last period (2009–2013), when we introduced induction therapy for patients with central tumors or with mediastinal nodal involvement, 38 of 651 who received surgery underwent induction therapy. The PN:SL ratios of those who did and did not undergo induction therapy were 15 (PN: 1, SL: 15) and 4.25 (PN: 8, SL: 34), respectively (*p* = 0.25).

**Table 4:** Numbers of surgeries by type during the study period

Procedures	1994–1998	1999–2003	2004–2008	2009–2013	Total
Total surgery	391	504	501	651	2047
Pneumonectomy	22 (5.63 %)	16 (3.17 %)	7 (1.40 %)	9 (1.38 %)	54 (2.64 %)
Induction therapy	0	1	0	1	2
Sleeve lobectomy	36 (9.20 %)	40 (7.94 %)	26 (5.20 %)	49 (7.53 %)	151 (7.38 %)
Induction therapy	0	0	3	15	18
PN:SL ratio	1.64	2.50	3.71	5.44	2.80

*PN* pneumonectomy, *SL* sleeve lobectomy

## Discussion

In the present study, we reviewed the evolution of a lung-sparing strategy for NSCLC over 20 years in a single institution. The ratio of PN among total surgeries decreased significantly, while the PN:SL ratio increased significantly over the study period. Induction therapy appeared to contribute to the increase in the PN:SL ratio. Long-term survival in patients with pN0–1 was significantly better in the SL group than in the PN group, and postoperative mortality and morbidity were significantly lower in the SL group than in the PN group.

The application of SL has been extended to patients with sufficient pulmonary function as a lung parenchyma-sparing strategy that aims to decrease postoperative mortality and morbidity and to improve long-term outcomes. The operative risk of PN is still high, with mortality rates ranging from approximately 5 to 10 % in recent reports, despite the improvements in surgical techniques and postoperative care [18–21]. In addition, in terms of quality of life, Balduyck et al. evaluated quality of life after SL and PN prospectively and concluded that SL offers better quality of life than does PN in terms of dyspnea, pain, and shoulder dysfunction [21]. Martin-Ucar and colleagues demonstrated in their prospective study of parenchymal-sparing lung surgery that the rate of PN could decrease significantly with increasing use of SL [22].

We have been trying to avoid PN by making full use of broncho-vascular reconstruction. The ratio of PN among total surgeries for NSCLC was 2.64 % throughout the study period, and it decreased to less than 1.5 % in the past 10 years. According to recent reports using the national database, the ratio of PN among surgeries for NSCLC was 7.2–12.3 % [18, 19, 23]. Even in the institutes aggressively pursuing SL, the ratio of PN was 4.3–17.6 % [22]. We speculate that one of the reasons for this low PN rate other than aggressive use of SL is the increase of small peripheral lung cancer in recent years, especially in Japan. Another reason would be the aggressive use of extended SL in recent years. In our study, extended SL was performed in 27 patients. Twenty-one of those 27 patients underwent extended SL in recent 10 years, and it appeared to contribute to decrease the PN substantially. Very few studies have reported the outcomes of extended SL [11, 12, 24, 25]. The number of patients who underwent extended SL in these reports ranged from 15 to 27.

Gómez-Caro and coworkers reported their aggressive policy of PN avoidance and showed that the PN:SL ratio can be used as a quality standard and that the ratio should be at least 1.5 or 2 [13]. Regarding the PN:SL ratio, the present study demonstrated an increased PN:SL ratio to more than 5 in the last period and the average was 2.8. This PN:SL ratio is extremely high, taking into consideration that the PN:SL ratio reported by Gómez-Caro and colleagues was 2.6, which was the highest in their review. The possible factors contributing to our high PN:SL ratio may be the use of induction therapy in addition to the decrease of PN which was described before. Since we introduced induction therapy for the patients with central tumors or tumors with pathologically proven clinical N2 in 2008, the PN:SL ratio increased dramatically. During the last 5 years, the PN:SL ratios of those who underwent induction therapy were higher than the ratios of those who did not, although the difference was not significant. Rendina et al. reported that only 5 of 68 patients (7.3 %) who received induction chemotherapy underwent PN, while 27 patients (39.7 %) underwent broncho-vascular reconstructive surgery, which results in a PN:SL ratio of 5.4 [16]. They suggested that the need for PN could be reduced by induction therapy while maintaining the same rates of radical treatment and survival. Since most of our cases that received induction therapy were observed for less than 5 years, we must follow these cases carefully.

One of the major concerns with SL is locoregional recurrence. According to the recent meta-analysis by Shi et al., the pooled locoregional recurrence with SL was 14.44 % compared with 26.08 % with PN, which was not statistically significant [8]. In our study, although data were only available for patients who underwent surgery in the past 11 years, the locoregional recurrence rates of SL and PN were 17.1 and 15.8 %, respectively, which are almost consistent with previous reports that showed similar local recurrence rates between SL and PN [1, 3, 6].

Our study has some limitations. It is retrospective, and it had a long time interval of patient recruitment that had changes in the treatment of lung cancer. In addition, the results of recurrence were only available in 52.2 % of patients. Another limitation is that since PN was significantly associated with advanced disease compared with SL, the difference in outcomes between groups was associated with selection bias. Therefore, our findings should be interpreted with caution.

In conclusion, this study demonstrated the actual evolution of a lung-sparing strategy over 20 years in a single institution. A lung-sparing strategy with SL could decrease the ratio of PN substantially, with less mortality. Induction therapy may facilitate SL and increase the PN:SL ratio.
